# Fracture behaviors under pure shear loading in bulk metallic glasses

**DOI:** 10.1038/srep39522

**Published:** 2016-12-23

**Authors:** Cen Chen, Meng Gao, Chao Wang, Wei-Hua Wang, Tzu-Chiang Wang

**Affiliations:** 1 State Key Laboratory of Nonlinear Mechanics, Institute of Mechanics, Chinese Academy of Sciences, Beijing 100190, China; 2 Institute of Physics, Chinese Academy of Sciences, Beijing 100190, China

## Abstract

Pure shear fracture test, as a special mechanical means, had been carried out extensively to obtain the critical information for traditional metallic crystalline materials and rocks, such as the intrinsic deformation behavior and fracture mechanism. However, for bulk metallic glasses (BMGs), the pure shear fracture behaviors have not been investigated systematically due to the lack of a suitable test method. Here, we specially introduce a unique antisymmetrical four-point bend shear test method to realize a uniform pure shear stress field and study the pure shear fracture behaviors of two kinds of BMGs, Zr-based and La-based BMGs. All kinds of fracture behaviors, the pure shear fracture strength, fracture angle and fracture surface morphology, are systematically analyzed and compared with those of the conventional compressive and tensile fracture. Our results indicate that both the Zr-based and La-based BMGs follow the same fracture mechanism under pure shear loading, which is significantly different from the situation of some previous research results. Our results might offer new enlightenment on the intrinsic deformation and fracture mechanism of BMGs and other amorphous materials.

The fracture behaviors of BMGs are more complicated than those of crystalline materials due to the unknown atomic structural and metallic bonding^1^,^2^, which is one of the toughest and hottest research directions in the field of material science. Under external loading, the stress-strain curves show that the BMGs usually exhibit the typical brittle fracture behaviors without obvious macroscopic plasticity. Meanwhile, it has been found that the plastic deformation in BMGs is highly localized into narrow shear bands (SBs)^3^. As shearing deformation proceeds, the friction heat and the drastically reduction of viscosity within the SBs strongly weaken the load capacity of BMGs, leading to the “work softening” behaviors and subsequently catastrophic fracture^4^. What is more, under the different loading conditions, BMGs display completely different fracture behavior^5^,^6^,^7^,^8^,^9^,^10^. For example, the compressive strength is always higher than the tensile strength, and the fracture plane deviates from the maximum resolved shear stress plane (45°), which is greater than 45° under tension and less than 45° under compression^5^,^6^,^7^,^8^. The fracture surface morphologies are also diverse under the different loading conditions^10^,^11^. It indicates that the stress state can greatly affect the macroscopic fracture behaviors and microscopic fracture mechanism. Also, the normal stress plays an important role in the deformation and fracture process^12^,^13^. These complicated fracture behaviors severely hinder the deeply understanding of the intrinsic deformation and fracture mechanism, which is of great importance for the scientific research and practical application for BMGs. Therefore, it is urging to search a suitable and efficient method to study the intrinsic mechanical behavior of BMGs.

On the other hand, for the traditional theories, it is a big challenge to be used to understand and predict the complicated fracture behaviors of BMGs, especially for the crystalline plasticity theory based on the defect concepts^14^. It is also found that the typical strength criteria, such as Von-mises and Tresca criteria, are not suitable for understanding the deviation of fracture plane from the maximum shear stress plane^8^, and the applicability of the Mohr-Coulomb criterion is still controversial for BMGs^8^,^15^,^16^. In the past several decades, many researchers have done a lot of work on the deformation and fracture mechanism for BMGs and all kinds of theories have been proposed^3^,^16^,^17^,^18^,^19^,^20^,^21^,^22^. For example, the shear transformation zone (STZ) theory^3^,^17^ assumes that a shear-induced atomic rearrangement occurs at local clusters that are a few to hundreds of atoms in size. The free volume theory^18^,^19^ takes into account the dependence of flow defect concentration on deformation and describes the relationship between the change of free volume and applied shear stress. In addition, some new fracture criteria have been set up to describe the fracture behaviors of BMGs^16^,^20^, a universal scaling law that demonstrated the intrinsic correlation between yielding and glass-liquid transition were reported for the understanding of strength and deformation of BMGs^21^, and the effect of thermal history on shear band initiation was investigated for the yield behavior in BMGs^22^. These new theoretical and experimental progresses have greatly promoted the research of mechanical behaviors for BMGs. However, there still exist some tough problems until now. For example, although the STZ theory and free volume model could be used to understand the mechanical behaviors, the normal stress effect is hard to be verified in the existing tensile and compressive fracture experiments^12^,^13^, and the intrinsic deformation and fracture mechanism under the pure shear stress field have not been made clear.

The pure shear fracture test is a unique mechanical method that ruling out the normal stress to study the intrinsic deformation and fracture mechanism for traditional materials^23^, which may be a suitable method to test the normal stress effects on the fracture behaviors and further understand the intrinsic fracture mechanism for the BMGs. For example, the exact value of the pure shear fracture strength is a necessary parameter for the establishment of strength theory. The pure shear fracture morphology and fracture angle can directly reflect the intrinsic fracture mechanism, and the normal stress effect can be easily displayed by comparing with the tensile and compressive fracture behaviors.

Some researchers have realized the irreplaceability and significance of pure shear fracture test for BMGs and made some efforts to it. The traditional torsion tests have been carried out to obtain the shear strength^15^. And the mode II fracture toughness of Zr-based BMGs has been carefully studied, such as the single edge notched flexure (ENF) fracture test designed by Flores and Dauskardt^24^, and the asymmetric four-point bend fracture test with the single notch specimen adopted by Ramamurty and coworkers^25^,^26^. While, some problems still exist in these pure shear test methods due to the limited glass forming ability and the complicated fracture behaviors of BMGs. The torsion test is usually carried out for metallic material by the thin-walled tubular specimens^23^, and the limited glass forming ability makes the cylinder specimen is the only choice for the torsion test of BMGs. Thus, the torsion test is obviously inappropriate for most of BMGs since the shear stress distributes linearly along the axial direction of the cylinder specimen. Meanwhile, for the ENF fracture test^24^ and the asymmetric four-point bend fracture test^25^,^26^, the narrow single notches are machined on the side of these specimens to obtain pre-cracks, which induced stress concentration near the notch tip. The introduction of pre-cracks makes the mentioned mode II fracture tests just suitable for the investigation of fracture toughness. Thus, all of the methods mentioned above are not suitable for the measurement of shear strength, which is a big challenging bottleneck.

In this work, we adopted a unique antisymmetrical four-point bend (anti-FPB) shear test method and specially designed the BMG specimens with two aligned 90° V-notches at the antisymmetrical center to realize a uniform pure shear stress field. This method was applied to systematically study the pure shear fracture behaviors at room temperature for two typical BMG systems of Zr_52.5_Cu_17.9_Ni_14.6_Al_10_Ti_5_ and La_60_Ni_15_Al_25_, which show completely different behaviors and mechanism in other conventional fracture tests. By carefully investigating the pure shear fracture strength, fracture surface morphology, it can be found that Zr- and La-based BMGs show little difference the macroscopic fracture behavior and microscopic fracture mechanism under pure shear loading, which indicates the common and intrinsic fracture mechanism in various BMGs. These results could be greatly helpful for deeply understanding the fracture behavior and deformation mechanism of BMGs and other amorphous materials.

## Results

### Pure shear stress-strain curves and shear fracture strengths

**Figure 1:**
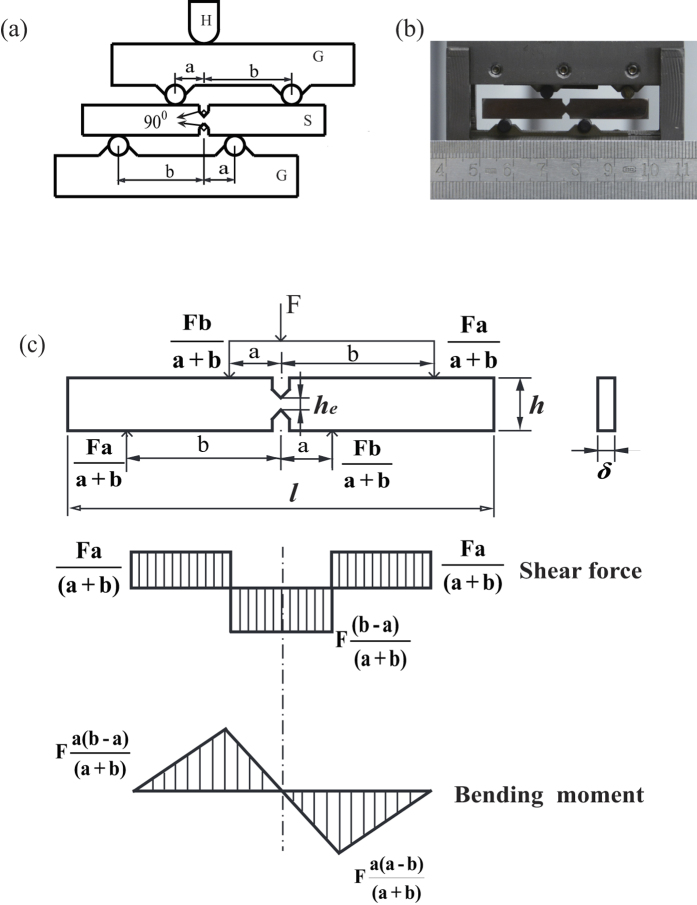
Anti-FPB pure shear test. (**a**) Schematic view of the experiment principle: *H* is the press head of the machine; *G* is the grooved block holding the loading rods; *S* is the specimen. (**b**) Test fixture. (**c**) Diagrams of loading configuration, shear force and bending moment for the specimen. *F* is the force of the machine head; *a* and *b* are distances from the antisymmetrical center to the loading points respectively; *h*_*e*_ is the effective height of the reduced cross-section; *δ* is the thickness of the specimen; *h* is the effective height of the specimen.

**Figure 2:**
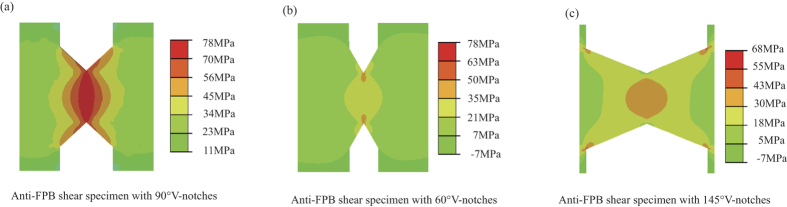
Shear stress distribution contour of finite element analysis for the anti-FPB shear specimen with aligned V-notches. (**a**) 90° V-notches. (**b**) 60° V-notches. (**c**) 145° V-notches.

As shown in the methods section, the anti-FPB shear test method is introduced in Fig. 1a and the BMG specimens with two aligned 90° V-notches at the antisymmetrical center are specially designed to realize the pure shear stress field (Fig. 1c). To further confirm that the distribution of the shear stress between the notch tips is uniform, we also use the finite element method to simulate the shear stress distribution for the anti-FPB shear specimen with different angles of the introduced V-notches in Fig. 2. The simulation results show that only the specimen with 90° V-notch display a uniform shear stress field, which confirm the justifiability of our anti-FPB shear test method. By applying this method, we investigate the pure shear fracture behaviors for Zr- and La-based BMGs.

**Figure 3:**
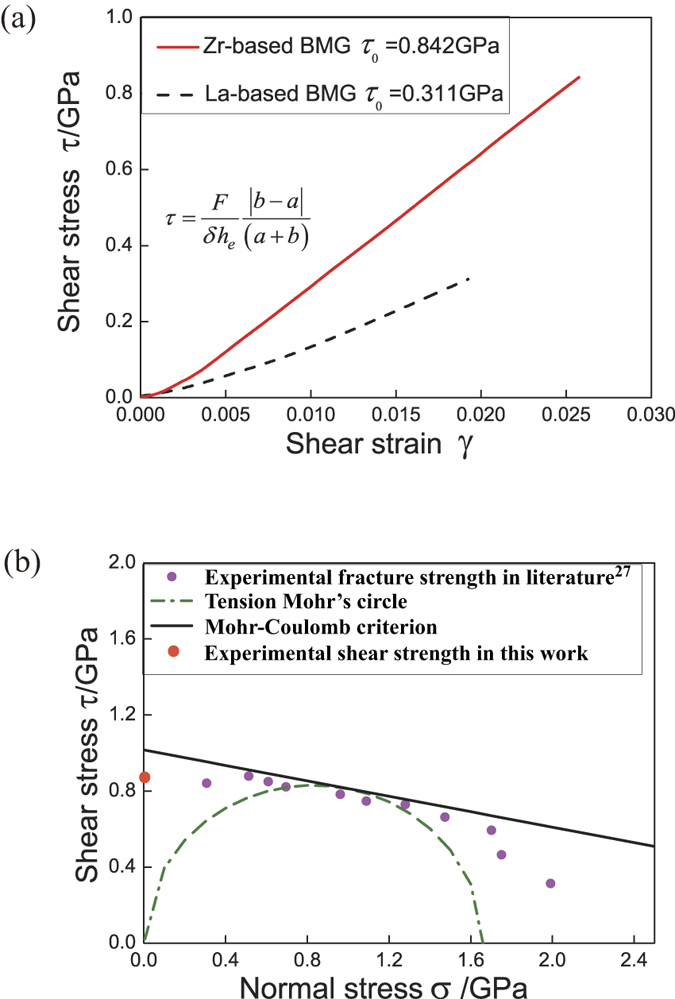
(**a**) Pure shear stress-strain curves for Zr-based and La-based BMGs. Shear stress *τ* is calculated by the inserted equation. (**b**) Comparison of the shear fracture strength between the experimental and calculated results by Morh-Coulomb criterion for Zr_52.5_Cu_17.9_Ni_14.6_Al_10_Ti_5_.

The detailed pure shear stress-strain curves of Zr- and La-based BMGs are shown in Fig. 3a. From Fig. 3a, it can be found that the fracture takes places immediately when the shear strain reaches about 2.5% for Zr-based BMGs and 2% for La-based BMGs, which display as the typical brittle materials without obvious macroscopic plasticity. According to the experimental principle of the anti-FPB shear test method, the shear stress along the notch tips axis is determined by the inserted equation in the Fig. 3a. Thus, the shear fracture strengths for Zr- and La-based BMGs are 0.842 GPa and 0.311 GPa, respectively.

The existing fracture criteria for BMGs largely depend on the tensile and compressive experimental results^27^, and their applicability for the pure shear fracture is needed to confirm by further experiments, such as the Mohr-Coulomb (M-C) criterion. Thus, we compared the experimental value of shear strength for Zr_52.5_Cu_17.9_Ni_14.6_Al_10_Ti_5_ with the calculated one by the M-C criterion. The M-C criterion can be described as 
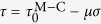
, where 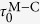 is the critical shear fracture stress, and *μ* is a constant which reflects the effect of the normal stress. For Zr_52.5_Cu_17.9_Ni_14.6_Al_10_Ti_5_, *μ* = 0.249 and the calculated value of 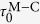 is 1.06GPa^27^. While the experimental shear strength is 0.842 GPa, which is obviously lower than the calculated results. As shown in Fig. 3b, the marked difference reflects that the M-C criterion is not suitable for the pure shear fracture behavior of BMGs. Therefore the fracture criterion for BMGs should be established more accurately based on pure shear strength in our work with tension and compression experimental data in the literature, which will be our focus in the future.

### Pure shear fracture angle

**Figure 4:**
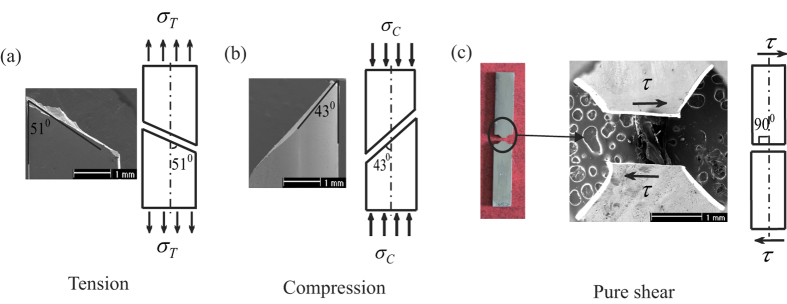
SEM images and schematic diagrams of fracture angle for Zr_52.5_Cu_17.9_Ni_14.6_Al_10_Ti_5_ under different loading modes. (**a**) Tension. (**b**) Compression. (**c**) Pure shear.

From the existing experimental results^5^,^6^,^7^,^8^, it can be found that the tensile and compressive fracture angles of BMGs deviate from the angle of the maximum resolved shear stress plane (45°). Generally speaking, the tensile fracture angles are more than 45°, and the compressive fracture angles are less than 45°. Figure 4 compared the fracture angles schemes for Zr_52.5_Cu_17.9_Ni_14.6_Al_10_Ti_5_MG specimens under uniaxial tension, uniaxial compression and pure shear loadings. The existing investigation displays that the fracture occurs along the shear plane that around 51° with respect to the tensile axis^27^ (Fig. 4a) and 43° with respect to the compressive axis^27^ (Fig. 4b). The normal stress applied on the fracture plane is very high (about 1 GPa) and plays an important role in the fracture process for tensile and compressive fracture^12^,^13^. Under the Anti-FPB loading (Fig. 4c), the fracture occurs along the pure shear plane, and the micrograph also exhibits a flat edge of the fracture plane, which further indicates that the normal stress effect is prevented and the pure shear stress field is actually realized.

### Pure shear fracture morphology

**Figure 5:**
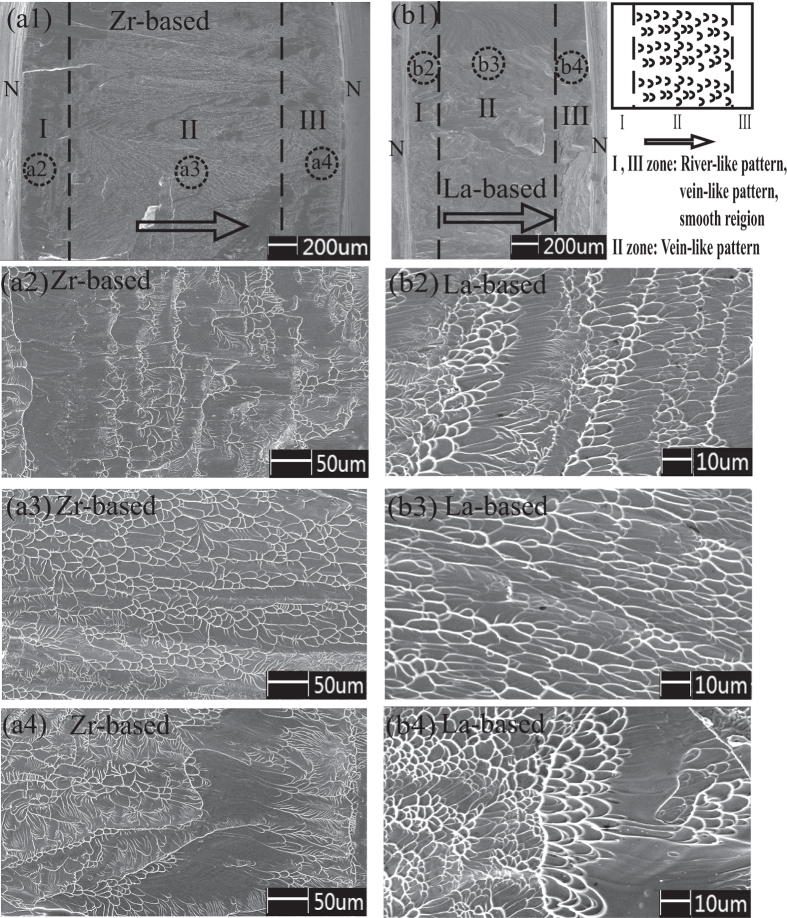
SEM images for pure shear fracture surface morphology of Zr_52.5_Cu_17.9_Ni_14.6_Al_10_Ti_5_ and La_60_Ni_15_Al_25_. (**a**) Zr_52.5_Cu_17.9_Ni_14.6_Al_10_Ti_5_. (**b**) La_60_Ni_15_Al_25_. (a1), (b1) are whole fracture morphology. Three characteristic zones are marked with I, II and III, and the schematic diagram is shown in the right part. Zone I and III stand for the parts near the notch edges and present mixed features; zone II presents the vein-like pattern. Arrows stand for the direction of shear Vein-like patterns. N represents the notch position. (a2), (a3), (a4) and (b2), (b3), (b4) are the detailed fracture morphology in zone I, II and III, respectively, which circled by the black dashed circles in Fig. 4 (a1) and (b1).

The pure shear fracture surfaces for the Zr- and La-based BMGs are displayed in Fig. 5. By comparing the detailed fracture morphology of Zr-based BMG in Fig. 5a1–a4 with that of La-based BMG in Fig. 5b1–b4, one can see clearly that the evolution of fracture morphology for Zr-based and La-based BMGs display the same trend. For these two kinds of BMGs, the whole morphology can be divided into three characteristic zones (zone I, II, III). The two zones at the edges of the fracture surface (zone I and III) present the complicated features with widths of 200~300 μm. Figure 5a2,a4 (Zr-based) and b2, b4 (La-based) show the detailed microscopic fracture morphology in zone I and III, which are consisted of smooth zones, river-like and vein-like patterns. Since the discontinuity of the material induced by the sample machining process are not be avoided completely at the notch edges, the changes of microstructures in the zones I and III result in the disorganization of the fracture features near these regions.

It is noted that the sample machining can not affect the main part of the fracture surface, and the pure shear stress still plays a leading role in the fracture plane. As the most important and dominated part on the whole surface, zone II displays regular shear-driven vein-like pattern feature for both Zr- and La-based BMGs (Fig. 5a3,b3). The veins spread over almost the whole surface except the region near the notch edge and arrange in an identical direction that exactly parallel to the direction of shear stress. The shear-driven vein-like pattern further confirms that the pure shear fracture is achieved by anti-SFPB shear test method, and the main features on the fracture surface remain same for the specimens with different notch sizes. The vein-like pattern feature also indicates that the Zr- and La-based BMGs follow the same fracture mechanism under pure shear loading.

**Figure 6:**
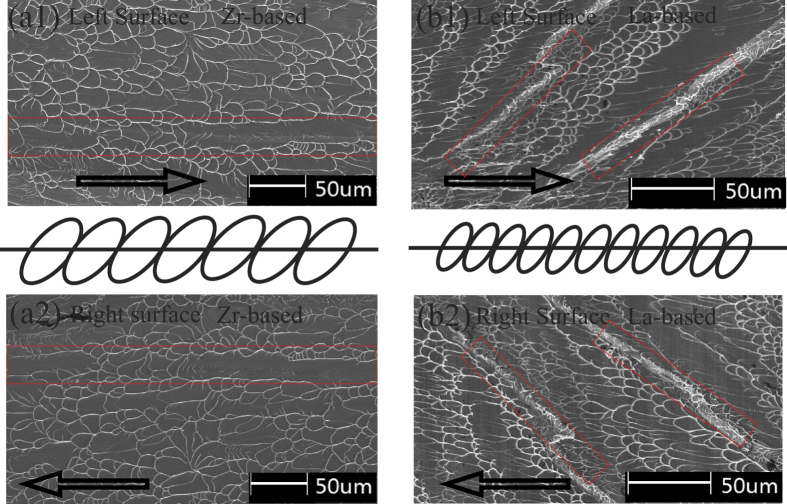
Fracture morphologies on the two corresponding fracture surfaces in the same sites for an identical specimen. (**a**) Zr_52.5_Cu_17.9_Ni_14.6_Al_10_Ti_5_. (**b**) La_60_Ni_15_Al_25_.

The two corresponding fracture surfaces of the specimens for Zr- and La-based BMGs are also compared by SEM. As illustrated in Fig. 6, the directions of shear-driven vein-like patterns are opposite on the two corresponding surfaces and the veins are matched in the same sites. On the other hand, the average size of vein-like pattern on fracture surface for Zr-based BMG is larger than that for La-based BMGs. From Table 1, it can be found that the poisson ratio and plasticity deformation under compression of Zr-based BMG are also higher than those of La-based BMG^28^, which is consistent with the previous investigation^4^,^29^, and the size of dimple (the size of the plastic zone at crack tip) increases with the increase of the poisson ratio for most BMGs.

## Discussions

**Figure 7:**
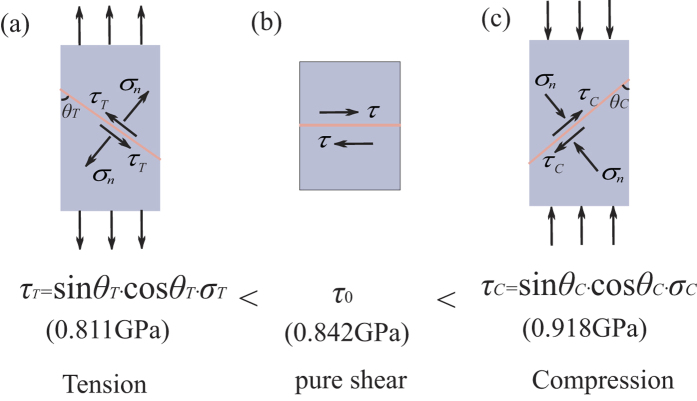
Comparison of shear stress along the fracture plane under different loading modes. (**a**) Tension. (**b**) Pure shear. (**c**) Compression.

The applied stress σ for tensile and compressive specimens of BMGs can be resolved into two components: the shear stress *τ* in the plane of flow, which makes the two parts of the specimen slide over each other, and the normal stress *σ*_*n*_, which is perpendicular to the plane of flow. Obviously, the normal stress *σ*_*n*_ tries to pull the two parts of the specimen apart under unaxial tension, but extrude them under unaxial compression. Under uniaxial tensile loading, the fracture resolved shear stress *τ*_*T*_ on the tensile fracture plane can be calculated by *τ*_*T*_ = sin*θ*_*T*_∙cos*θ*_*T*_∙*σ*_*T*_, where *σ*_*T*_ is uniaxial tensile strength and *θ*_*T*_ is tensile fracture angle (Fig. 7a). Under uniaxial compressive loading, the fracture resolved shear stress *τ*_*C*_ on the compressive fracture plane is *τ*_*C*_ = sin*θ*_*C*_∙cos*θ*_*C*_∙*σ*_*C*_, where *σ*_*C*_ is uniaxial compressive strength, and *θ*_*C*_ is compressive fracture angle (Fig. 7c). For Zr_52.5_Cu_17.9_Ni_14.6_Al_10_Ti_5_, *θ*_*T*_ = 51°, *σ*_*T*_ = 1.66GPa^12^, *τ*_*T*_ = 0.811 GPa; *θ*_*C*_ = 43°, *σ*_*C*_ = 1.84GPa^12^, *τ*_*C*_ = 0.918 GPa. Thus, the pure shear strength *τ*_0_ (0.842 GPa) satisfies that: *τ*_*T*_ < *τ*_0_ < *τ*_*C*_. The tensile normal stress makes the specimen easier to fracutre at lower resolved shear stress. On the contrary, the obstruction of compressive normal stress makes the final fracture occur at higher resolved shear stress.

From the existing investigation, it can be found that Zr-based and La-based BMGs show different fracture behavior in other conventional fracture tests, especially the mode I fracture. Firstly, the fracture toughness of Zr-based and La-based BMGs are about 20 MPa m^1/2^ and 7 MPa m^1/2^, respectively (Table 1). Moreover, the fracture mechanisms of Zr-based and La-based BMGs are different in mode I fracture^11^. For the Zr-based BMG, the dimple pattern on the fracture surface^11^ indicates a ductile fracture mechanism, which initiates from the fluid meniscus instability mechanism and the crack grows inside a dominant shear band^30^. For the La-based BMG, the nanocorrugation pattern on the fracture surface^10^,^11^ indicates a brittle fracture mechanism, which propagates from the nanoscale void nucleation and coalescence^10^,^31^. These two competing fracture mechanisms have been investigated by experiments^10^,^11^, theoretical analysis^30^,^31^ and dynamic simulation^32^,^33^. Therefore, despite the macroscopic fracture behavior of BMGs display as brittle material, the BMGs have been divided into two classes based on the microscopic fracture mechanisms in the existing investigation: ductile and brittle BMGs. It is obviously that Zr-based BMG is ductile and La-based BMG is brittle from microscopic view.

**Figure 8:**
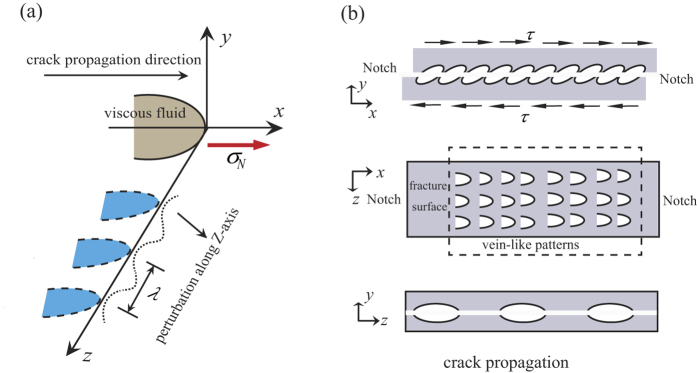
Fracture mechanism of BMGs under pure shear loading. (**a**) Phenomenal model of fracture morphology formation during the pure shear stress based in the fluid meniscus instability (FMI) at the crack tip. (**b**) The formation scheme of vein-like patterns.

From the experimental results in this work, it can be found that the ductile Zr- and brittle La-based BMGs show little qualitative difference in pure shear fracture behaviors, which is different with the investigations of mode I fracture mentioned above. Especially the vein-like pattern, which is considered to a direct reflection of the ductile fracture mechanism^33^, is dominated on the pure shear fracture surface for both ductile Zr- and brittle La-based BMGs. Our results reveal that the normal stress has a great influence on fracture mechanism of BMGs. The pure shear fracture test, which rules out the normal stress effect, displays that although the macroscopical fracture of BMGs behaves as brittle material, the intrinsic fracture mechanism of BMGs follow the ductile mode. The existing investigation of ductile fracture mechanism for BMGs shows that the crack occurs in the dominant shear band and eventually leads to the fracture of the specimen^25^. At the same time, the temperature rise in the shear band, which has been verified by experiment, is found to be closely related to the formation of veins^34^. The pure shear fracture, which occurs catastrophically along the shear plane without any geometric constraint, would make the elastic energy within samples release instantaneously and cause a significant temperature rise in the dominant shear band. And the temperature rise during the fracture process can be roughly estimated if the elastic energy release is assumed to be fully converted to the heat in the fracture plane^34^,^35^, which can be calculated by 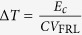, where, *Εc* is total elastic energy, *C* is the specific heat per unit volume, *V*_*FRL*_ is the volume of fracture relevant layer. 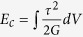, where τ is shear stress, G is shear modulus, *V* is the volume of the specimen. For Zr_52.5_Cu_17.9_Ni_14.6_Al_10_Ti_5_BMG, the shear fracture strength τ_0_ = 0.842 GPa, the shear modulus *G* = 37.4 GPa, the total elastic energy is about 4 J, *C* = 3 J/(K∙cm^3^), *V*_*FRL*_ = 0.2 cm × 0.2 cm × 0.9 × 10^−4^ cm (the thickness of the fracture relevant layer for Zr-BMG is about 0.9 um^13^). According to the above calculation, the estimated temperature rise is above thousands of degree, which is enough to reach the melting point (1075 K for the present Zr-based BMG) and causes the melting of the glassy phase in the fracture layer. Since the temperature rise makes the amorphous phase melt in the fracture layer, the glassy phase at crack tip can be treated as a viscous fluid. Therefore, we propose a phenomenal model based on the fluid meniscus instability (FMI)^36^ mechanism to understand the pure shear fracture behaviors, as shown in Fig. 8a. Due to the balance between the surface tension of the viscous fluid and the negative pressure effect generated by the local stress, the viscous fluid would be shaped as a meniscus at crack tip and move along the X-axis. Meanwhile, the meniscus becomes unstable when there appears a perturbation along Z-axis. Argon’s analysis showed that when the wave length of perturbation *λ* satisfied that 
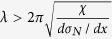
 (*χ*: surface energy, 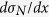: pressure gradient along the X-axis)^36^, the perturbation along Z-axis would develop into a finger shape, and the finger shape would penetrate the BMGs with the movement of the crack tip and form a stable vein-like pattern on the fracture surface when the complete fracture happens.

Based on the above analysis of the FMI theory at crack tip^36^, the significant temperature rise and large negative pressure gradient along the crack propagation direction are two indispensable ingredients, which result in the formation of a vein-like pattern. In the shear band, the viscous fluid caused by temperature rise is shaped as a meniscus (the upper one in Fig. 8b). Meanwhile, the perturbation would render the viscous fluid meniscus unstable along the direction perpendicular to crack propagation (Z-axis) and make them develop to a finger shape (the lower one in Fig. 8b). When the specimen crack completely, the vein-like patterns take shape along the shear slip direction on the fracture surface due to the formation of finger shaped viscous fluids (the center one in Fig. 8b). The arrangement direction of the veins is along the shear stress direction.

## Conclusion

The Anti-FPB test method is applied to study the pure shear fracture behaviors of the BMGs. For the plate-shaped specimens of Zr_52.5_Cu_17.9_Ni_14.6_Al_10_Ti_5_ (ductile) and La_60_Ni_15_Al_25_ (brittle) BMGs, the pure shear fracture happens immediately when the plastic deformation appears, and the shear fracture strengths are obtained to be 0.842 GPa and 0.311 GPa, respectively. The pure shear strength *τ*_0_ of Zr_52.5_Cu_17.9_Ni_14.6_Al_10_Ti_5_ BMG satisfy that: sin*θ*_*T*_∙cos*θ*_*T*_∙*σ*_T_ < *τ*_0_ < sin*θ*_*C*_∙cos*θ*_*C*_∙*σ*_C_ (*σ*_T_: uniaxial tensile strength; *θ*_T_: tensile fracture angle; *σ*_C_: uniaxial compressive strength; *θ*_C_: compressive fracture angle), which is determined by the normal stress effect. The pure shear fracture morphologies for these two kinds of BMGs are found to be dominated by the vein-like patterns with a unified direction, which implies the ductile fracture mechanism. To further analyze the pure shear fracture mechanisms of BMGs, we propose a phenomenal model for the pure shear fracture mechanism based on FMI theory, which shows that the pure shear fracture morphology formation is mainly dependent on the temperature rise in the main shear band and the large negative pressure gradient along the crack propagation direction. The experimental results of pure shear fracture test show that although the stress-strain curves of BMGs behave as the typical brittle materials in macroscopical view, the vein-like patterns on the fracture surfaces indicate the same ductile fracture mechanism for different kinds of BMG systems. These new enlightenment from the pure shear fracture test would help us to further understand the complicated behavior of BMGs.

## Methods

### Experiment principle

The anti-FPB shear test is an effective method for measuring shear strength and shear modulus of brittle materials^37^,^38^. As shown in Fig. 1a, the four loading rods, which are embedded into the grooved blocks, are placed anti-symmetrically in the plane for a plate specimen. To avoid slip in the loading process, the grooved blocks are fixed into the work-holding (Fig. 1b). The illustration in Fig. 1c shows that the force is transmitted to the loading rod from the press head of the machine, which renders the anti-symmetrical four point loading mode for the plate specimen. The shear force and bending moment diagrams reveal that the bending moment is zero at the center of anti-symmetrical loading points, which gives pure shear-stress field in the cross section. All tests were performed under displacement control with a displacement rate of 0.2 mm/min. The distances from the antisymmetrical center to the loading points were *a* = 6 mm and *b* = 18 mm.

### Specimen preparation and characterization

The typical Zr-based metallic glass of Zr_52.5_Cu_17.9_Ni_14.6_Al_10_Ti_5_ (Vit 105) and La-based metallic glass of La_60_Ni_15_Al_25_ were chosen as model system due to their good forming ability and excellent mechanical properties. The plate-shaped specimens with geometric size of *l* = 50 mm, *h* = 6 mm, δ = 2 mm were prepared by induction melting a mixture of pure metal elements and then cast into Cu mold. For each specimen, the amorphous structure was confirmed by X-ray diffraction and differential scanning calorimetry (DSC). To get a uniform distribution shear stress field, the specimens were machined with two aligned 90° V-notches at the anti-symmetrical center (Fig.1a and c)^39^. This method had been verified to be effective for pure shear test by photoelastic and finite element analysis^40^. There were two different depth of the 90° V-notches notch adopted: 2.0 mm (*h*_*e*_ = 2 mm), and 2.25 mm (*h*_*e*_ = 1.5 mm) in this work. All specimens surfaces were carefully polished with 0.15 um diamond sandpaper.

To verify the effect of the angle of the introduced V-notches on the uniform pure shear stress field, the shear stress distribution of anti-FPB shear specimens with two aligned 90°, 60°, 145° V-notches were analyzed by finite element method (Fig. 2). In our simulation, the planar four nodes element was adopted, and the total number of elements for the whole model was 118667. The element sizes at the notch tips and between the two notches tips were about 0.001 mm. The simulation results shows that the stress concentration appears near the notch tips for the 60° and 145° V-notches specimens and the shear stress distributes uniformly for the 90° V-notch specimen. So the anti-FPB shear specimen with two aligned 90° V-notches in our work is effective for measuring the pure shear fracture strength.

## Additional Information

**How to cite this article**: Chen, C. *et al*. Fracture behaviors under pure shear loading in bulk metallic glasses. *Sci. Rep.* **6**, 39522; doi: 10.1038/srep39522 (2016).

**Publisher's note:** Springer Nature remains neutral with regard to jurisdictional claims in published maps and institutional affiliations.

## Figures and Tables

**Table 1:** Data of Young’s modulus *E*, Poisson ratio *ν*, fracture toughness *K*_*C*_ and plastic strain*ε*_*c*_ under compression loading for Zr- and La-based MGs used in this work^12^,^28^.

Metallic glasses	Young modulus *E* (GPa)	Poisson ratio *ν*	Fracture toughness *K*_*C*_ (MPa m^1/2^)	Plastic deformation under compression *ε*_*c*_(%)
Zr_52.5_Cu_17.9_Ni_14.6_Al_10_Ti_5_	88.6	0.37	20	5
La_60_Ni_15_Al_25_	47.5	0.34	7	0
